# Fast cycling of intermittent hypoxia in a physiomimetic 3D environment: A novel tool for the study of the parenchymal effects of sleep apnea

**DOI:** 10.3389/fphar.2022.1081345

**Published:** 2023-01-12

**Authors:** Alicia Jurado, Anna Ulldemolins, Helena Lluís, Xavier Gasull, Núria Gavara, Raimon Sunyer, Jorge Otero, David Gozal, Isaac Almendros, Ramon Farré

**Affiliations:** ^1^ Unitat de Biofísica i Bioenginyeria, Facultat de Medicina i Ciències de la Salut, Universitat de Barcelona, Barcelona, Spain; ^2^ Neurophysiology Laboratory, Department of Biomedicine, School of Medicine, Institute of Neurosciences, University of Barcelona, Barcelona, Spain; ^3^ Institut Investigacions Biomèdiques August Pi Sunyer, Barcelona, Spain; ^4^ The Institute for Bioengineering of Catalonia (IBEC), The Barcelona Institute of Science and Technology (BIST), Barcelona, Spain; ^5^ CIBER de Enfermedades Respiratorias, Madrid, Spain; ^6^ Department of Child Health, The University of Missouri School of Medicine, Columbia, KY, United States

**Keywords:** obstructive sleep apnea, hypoxia, cell culture, hydrogels, tissue slice, 3D culture, oxygen diffusion, disease model

## Abstract

**Background:** Patients with obstructive sleep apnea (OSA) experience recurrent hypoxemic events with a frequency sometimes exceeding 60 events/h. These episodic events induce downstream transient hypoxia in the parenchymal tissue of all organs, thereby eliciting the pathological consequences of OSA. Whereas experimental models currently apply intermittent hypoxia to cells conventionally cultured in 2D plates, there is no well-characterized setting that will subject cells to well-controlled intermittent hypoxia in a 3D environment and enable the study of the effects of OSA on the cells of interest while preserving the underlying tissue environment.

**Aim:** To design and characterize an experimental approach that exposes cells to high-frequency intermittent hypoxia mimicking OSA in 3D (hydrogels or tissue slices).

**Methods:** Hydrogels made from lung extracellular matrix (L-ECM) or brain tissue slices (300–800-μm thickness) were placed on a well whose bottom consisted of a permeable silicone membrane. The chamber beneath the membrane was subjected to a square wave of hypoxic/normoxic air. The oxygen concentration at different depths within the hydrogel/tissue slice was measured with an oxygen microsensor.

**Results:** 3D-seeded cells could be subjected to well-controlled and realistic intermittent hypoxia patterns mimicking 60 apneas/h when cultured in L-ECM hydrogels ≈500 μm-thick or *ex-vivo* in brain slices 300–500 μm-thick.

**Conclusion:** This novel approach will facilitate the investigation of the effects of intermittent hypoxia simulating OSA in 3D-residing cells within the parenchyma of different tissues/organs.

## 1 Introduction

**FIGURE 1:**
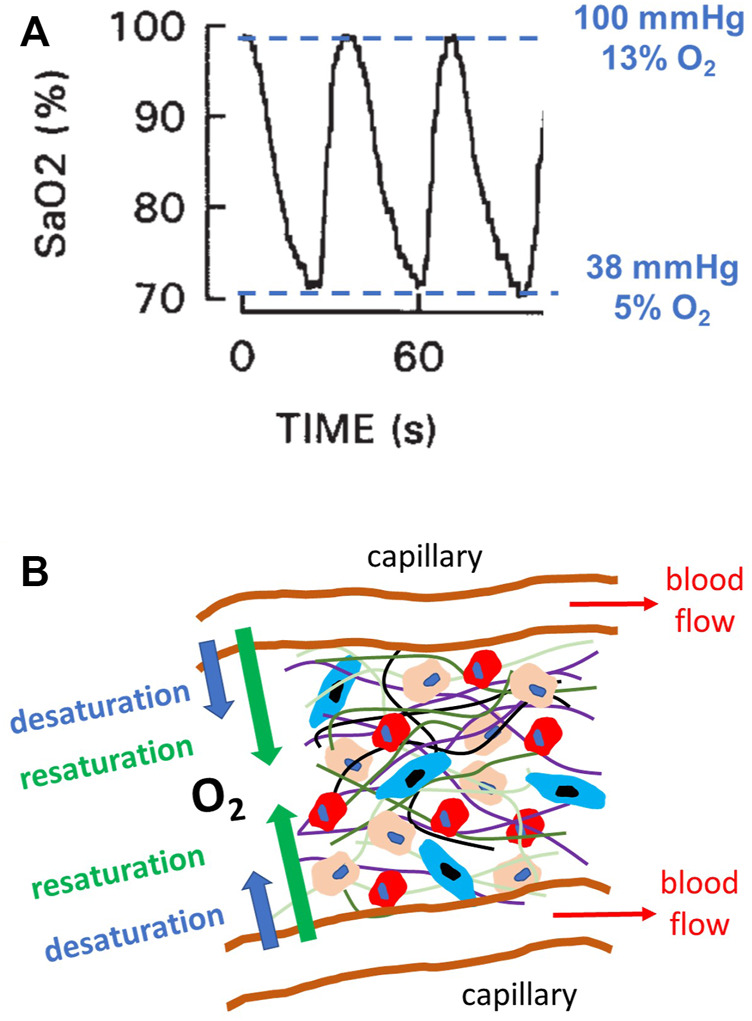
Intermittent hypoxia in obstructive sleep apnea (OSA). **(A)** Example of a recording of arterial oxygen saturation (SaO_2_) measured by pulse oximetry in a patient with severe OSA, showing the fast and marked hypoxemic events characterizing this disease. The figure also indicates the values of O_2_ partial pressure, and thus O_2_ percentage (for 760 mmHg atmospheric pressure), corresponding to the maximum and minimum levels of SaO_2_. Modified from Reference (Farré et al., 1998) and reproduced with permission. **(B)** Diagram representing a section of the parenchymal cells in any organ/tissue where different cell types (represented by different sizes and colors) reside within an extracellular matrix (different fibers represented by different color lines). This 3D environment is perfused by O_2_ from the arterial blood circulating through systemic capillaries. Given the intermittent hypoxemia, the magnitude of O_2_ diffusion through the capillary wall cycles (desaturation and resaturation), and cells are subjected to intermittent hypoxia caused by hypoxemic events **(A)**.

Obstructive sleep apnea (OSA) is a very prevalent respiratory disorder affecting patients of all ages, from children to the elderly (Benjafield et al., 2019). Patients suffering from OSA exhibit an abnormally increased collapsibility of the upper airway during sleep and thereby experience recurrent events of upper airway obstruction, usually terminated by an arousal. In more severe instances, these patients can sustain more than one obstructive event per minute of sleep. In addition to the consequences directly caused by the disruption of sleep architecture (diurnal somnolence, fatigue, increased traffic and labor accidents, poor quality of life, cognitive deficits, and depression), patients with OSA are also at increased risk of both morbidity and mortality from cardiovascular, metabolic, neurocognitive and malignant diseases (Sánchez-de-la-Torre et al., 2013; Gileles-Hillel et al., 2016; Gozal et al., 2020; Brockmann and Gozal, 2022). These adverse outcomes are primarily caused by the recurrent events of hypoxemia induced by the upper airway obstructions (i.e., apneas and hypopneas). Indeed, during these events, the absence/reduction of pulmonary alveolar ventilation results in transient reductions in the partial pressure of O_2_ in arterial blood, which is clinically assessed by non-invasively measuring arterial oxygen saturation (SaO_2_) by pulse oximetry (Figure 1A) (Farré et al., 1998). The recurrently hypoxic blood leaving the lungs enters the systemic capillaries that perfuse all the patient tissues and organs (Almendros et al., 2011; Reinke et al., 2011; Torres et al., 2014; Moreno-Indias et al., 2015), and this reduced O_2_ tension then diffuses into the surrounding extra capillary space, thereby subjecting parenchymal cells to intermittent hypoxia of varying degrees (Figure 1B). It has been well established that this noxious challenge triggers cascades of oxidative stress, inflammation, and immune and hormonal deregulations, which ultimately result in the increased end-organ and systemic adverse consequences of OSA (Sánchez-de-la-Torre et al., 2013; Gileles-Hillel et al., 2016; Gozal et al., 2020; Brockmann and Gozal, 2022).

Given that intermittent hypoxia is a major driver of the OSA-induced increase in morbidity and mortality, considerable experimental research efforts have been devoted to investigating how different types of cultured cells respond to these hypoxic cycles (Farré et al., 2018). However, a particular challenge in precisely achieving this research aim is to ensure that the cultured cells of interest are *de facto* subjected to the high-frequency events of hypoxia (up to 60 cycles per hour) characterizing OSA. Indeed, given the relatively slow process of O_2_ diffusion in water or other liquids, using the conventional procedure based on cyclically modifying the O_2_ partial pressure in the air on top of the culture medium is sub-optimal to achieve adequate fast changes of O_2_ partial pressure at the cell culture level (Allen et al., 2001). Fortunately, optimized experimental settings based on culturing cells on a thin O_2_-permeable membrane have been described to ensure that the cultured cells experience the desirable fast OSA-mimicking intermittent hypoxia events (Campillo et al., 2016; Minoves et al., 2017). However, these improved settings were designed and tested in cells cultured in 2D set-ups. Whereas such geometry is suitable for cells naturally living in monolayers (e.g., epithelial and endothelial cells), it is suboptimal when investigating the effects of intermittent hypoxia in most parenchymal (or tumor) cell types that naturally reside and evolve in a 3D environment within an extracellular matrix (ECM). To date, it has been a common approach to use 2D culture plates to investigate the biology of cells that naturally live *in vivo* in 3D microenvironments. However, there is solid emerging evidence that 2D and 3D cell microenvironments differently modulate essential cell functions (Duval et al., 2017; Jensen and Teng, 2020; Kusuma et al., 2022). Therefore, optimal study of parenchymal cell biology requires realistic simulation and replication of the native 3D configuration by culturing cells inside hydrogels or using *ex-vivo* precision-cut tissue slices (Tibbitt and Anseth, 2009; Caliari and Burdick, 2016; Dewyse et al., 2021). Accordingly, the present study aimed to devise and characterize a procedure for applying physiomimetic intermittent hypoxia to cells in a 3D microenvironment.

## 2 Materials and methods

**FIGURE 2:**
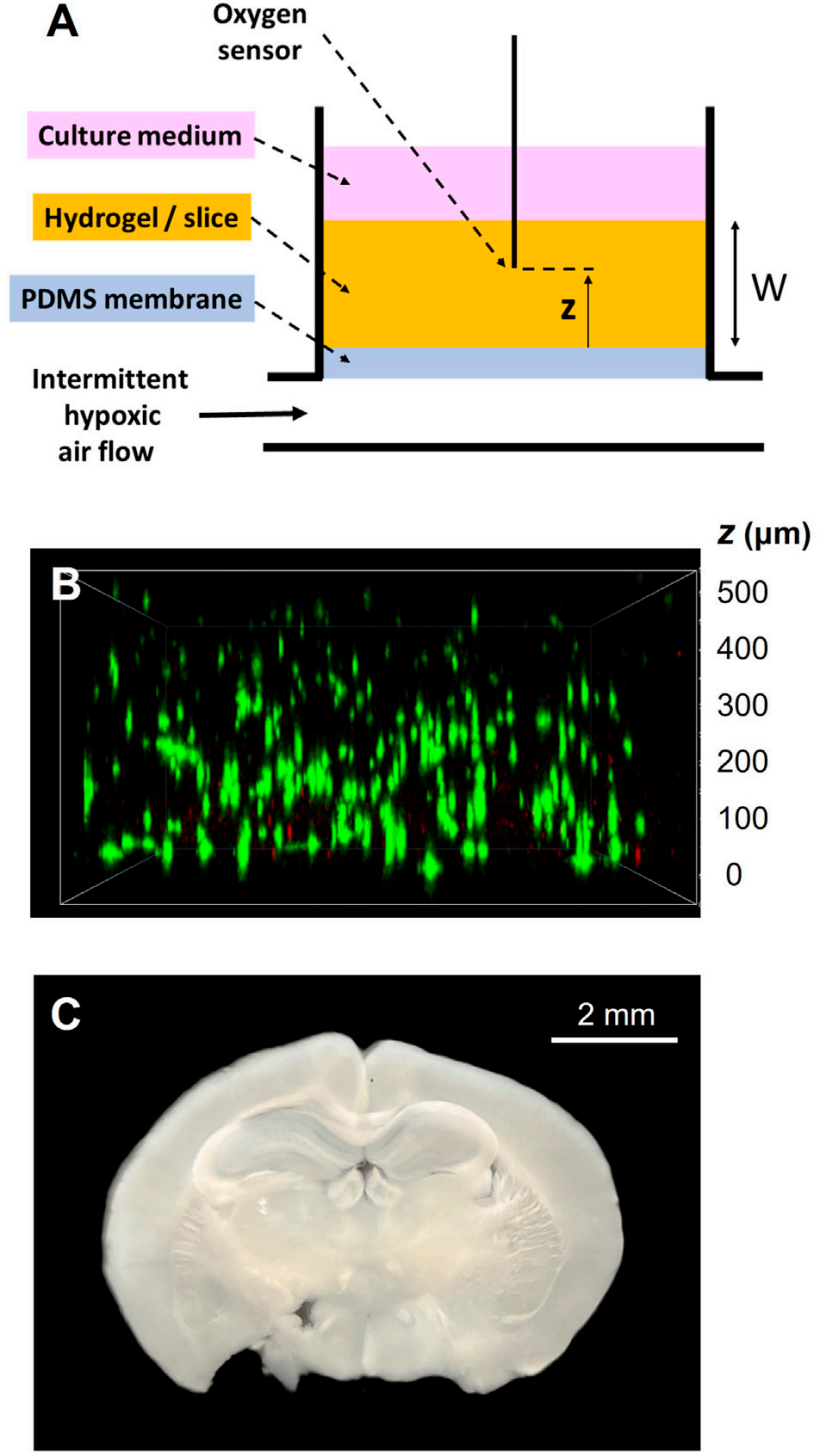
**(A)** Experimental setting for applying intermittent hypoxia to 3D-cultured cells. A culture well has a bottom consisting of a polydimethylsiloxane (PDMS) membrane. The compartment beneath the membrane is circulated with intermittent hypoxic air. The cells to be subjected to intermittent hypoxia are cultured within a 3D hydrogel or tissue slice of width W placed on the membrane. Only to characterize the setting, a thin fast-response O_2_ sensor probe is used to measure O_2_ concentration at different positions (*z*) across the sample. **(B)** Example of the achieved distribution of lung mesenchymal stromal cells within a lung ECM hydrogel (20 mg/mL) along the sample thickness, with green and red colors indicating live and dead cells respectively (Live/Dead viability kit; Invitrogen). Reproduced from Reference (Falcones et al., 2021) under Creative Commons Attribution (CC BY) license. **(C)** Example of a mouse brain slice to be subjected to intermittent hypoxia.

The experimental setting devised to apply intermittent hypoxia to cells in a 3D environment is schematically shown in Figure 2A. It is based on a previously described well with a bottom consisting of a membrane of O_2_-permeable polydimethylsiloxane (PDMS) (Campillo et al., 2016). A flow of intermittently hypoxic air at a frequency characterizing severe OSA (e.g., 60 apneas/h: 30 s of normoxic gas (i.e., air) and 30 s of hypoxic gas (i.e., target FiO2 of interest) circulates through the compartment beneath the membrane. The 3D scaffold (thickness W) containing the cells subjected to intermittent hypoxia (hydrogel made of natural ECM or precision-cut tissue slices) is placed on the PDMS membrane. A micrometric fast-response O2 sensor is only used to characterize the setting to measure O_2_ inside the 3D sample at different distances from the membrane (*z*, Figure 2A).

### 2.1 Well fabrication

As described in detail elsewhere (Campillo et al., 2016), the PDMS wells were assembled from 2 different parts: the upper one, composed of 6 wells constituting the culture chamber, and the bottom part with also 6 wells which comprised the gas chamber. Both parts were separated by a 165 μm thick gas-permeable PDMS membrane (Gel-Pak, Hayward, CA, United States). Each part of the setting was fabricated with a 1:10 mixture of curing agent and pre-polymer (Sylgard 184 kit, Dow Corning) using negative molds designed with the Ultimaker Cura software (Ultimaker, Utrecht, Netherlands) and printed with an Ultimaker S5 3D printer (Ultimaker, Utrecht, Netherlands) in polycarbonate material. In the center of the chip, an inlet tube connected to a servo-controlled gas blender (McQ, Virginia, United States) which was controlled by the Software Gas Mixture Creator (McQ, Virginia, United States) to provide the pre-defined specific gas mixture into the gas chamber. For the correct adhesion of the hydrogels, PDMS membranes were activated by placing the chips in a plasma cleaner (PDC-002, Harrick Scientific Products Inc. Pleasantville, NY) at maximum voltage for 1 min 90 s. Next, membranes were incubated with APTES 10% for 60 min and 5 mM genipin (Challenge Bio Products Co., Taiwan) for 45 min. Following each incubation, 2 washes of PBS 1X for 5 min were done. Lastly, the device was allowed to dry overnight.

### 2.2 Cell culture

Primary Rat Bone Marrow-derived Mesenchymal Stem Cells (rBMMSCs) acquired from Merck (SCR027) were used. Cells were expanded and cultured in MEM-α medium (Gibco) supplemented with 10% FBS and 1% Penicillin/Streptomycin, which was replaced every 48–72 h. At 80–90% of confluency, cells were trypsinized with TripLE express trypsin (Gibco) for 5 min and counted for hydrogel seeding. All experiments were performed with rBMMSCs at passage 4.

### 2.3 Hydrogel preparation

Porcine lungs were purchased in a local slaughterhouse and decellularized as previously reported (Falcones et al., 2021) based on a protocol previously described (Pouliot et al., 2016). In brief, pig lungs were perfused *via* the trachea and the vasculature with .1% Triton X-100 and 2% sodium deoxycholate for 24 h at 4°C. Afterwards, another perfusion with 1 M NaCl and DNase solution for 1 h was performed (4°C). Then, decellularized tissue was cut in small pieces and frozen at −80°C, lyophilized (Telstar Lyoquest55 Plus, Terrassa, Spain) and cryomilled (6,755, SPEX, Metuchen, NJ, United States) resulting in a fine powder. The lung ECM powder obtained was digested at 20 mg/mL concentration with pepsin from porcine gastric mucosa in .01 M HCl solution (1:10 concentration) under constant agitation at room temperature for 16 h. Then, the pH solution was adjusted to 7.4 (±.2) by adding 0.1 M NaOH and 10X PBS. The resulting pre-gel was stored frozen at −80°C for subsequent use. For preparing acellular hydrogels, lung ECM pre-gel at a concentration of 20 mg/mL was pipetted into the well and left to gellify at 37°C for 20 min followed by the addition of 1 mL of 1X PBS. This procedural approach allowed consistent and reproducible fabrication of hydrogel layers that were very uniform and of accurate thickness. For instance, when targeting a 500 μm-thick gel made of lung ECM, the achieved thickness measured by microscopy in 4 different random sites of the hydrogel surface in 4 different wells was 512 μm, with intra-sample and inter-sample variances of 1.7% and 1.6%, respectively. In the case of cell-laden hydrogels, rat bone marrow mesenchymal stem cells at two different concentrations (3 × 10^5^ cells/mL and 4.5 × 10^5^ cells/mL) were mixed with the lung ECM pre-gel. Then, the chip was placed at 37°C for 20 min to allow gelation prior to the addition of 1 mL of supplemented MEM-α medium. Cells were allowed to settle for 24 h in the incubator (20% O_2_, 5% CO_2_, 37°C) before starting the experiments.

### 2.4 Preparation of precision-cut mouse brain slices

Brain slices were obtained from 6-week-old C57BL/6J mice housed at the animal facility of the Medical School of the University of Barcelona. Mice were decapitated after being deeply anesthetized with inhaled isoflurane, the brain was immediately extracted with dissection tools and placed into the ice-cold artificial cerebrospinal fluid (aCSF) denominated as aCSF1 to reflect its use during brain tissue sectioning. Of note, the harvested brain was placed in aCSF1 within less than 1 min after sacrificing the animal. The aCSF1 used for slicing contained the following (in mM): 25 Sucrose, 2.5 Glucose, 125 NaCl, 2.5 KCl, 1.25 NaH_2_PO_4_, 26 NaHCO_3_, 5.9 MgCl_2_, adjusted to pH 7.3, osmolarity 320 mOsmol/kg, and bubbled with carbogen (95% O_2_, 5% CO_2_). The brain was glued to the cutting chamber of the slicer with cyanoacrylate glue in the proper orientation to prepare coronal cortico-hippocampal slices. Thick slices (300 and 500 μm) from the brain were prepared using a vibrating microtome (Leica VT1000 S), with a cutting blade vibrating at a 60 Hz frequency and moving at .125 mm/s forward speed. The slices were incubated in aCSF solution designed for recovery (aCSF2) at 34°C for 30 min, consisting of (in mM): 20 Glucose, 125 NaCl, 2.5 KCl, 1.25 NaH_2_PO_4_, 26 NaHCO_3_, 2 CaCl_2_, 1 MgCl_2_, adjusted to pH 7.4, osmolarity 310 mOsmol/kg, and continuously bubbled with carbogen. Afterward, slices were transferred with a glass Pasteur pipette to the measurement well, filled with aCSF2 and oxygenated with carbogen, in a thermostatic chamber at 37°C. The slice was held down by a nylon mesh attached to a platinum U-wire, which provided mechanical stability during the experiment. Animal care and procedures were approved and conducted following the CEEA-UB (Ethical Committee for Animal Research) from the University of Barcelona following European (2010/63/UE) and Spanish (RD 53/2013) regulations about the use and care of experimental animals.

### 2.5 Measurement of O_2_ concentration within the 3D sample

An optical fiber oxygen microsensor (OXR50, Pyroscience, Aachen, Germany) with a ≈40 µm sharp tip and a typical response time for 90% signal change <2 s was calibrated following the manufacturer’s instructions and attached to a specifically designed holder that allowed for micrometric-resolution vertical positioning. The signal from the oxygen sensor was recorded by an oxygen meter (FireStingO2; PyroScience) and digitally stored for subsequent analysis. This meter also carried out automatic temperature compensation by using the reference signal from a shielded submersible temperature sensor (TSUB36; PyroScience) placed into the culture well. Measurements were performed with the sensor tip introduced inside the sample (Figure 2A), at room temperature (23°C) in the case of acellular hydrogels and 37°C in cell-seeded hydrogels or tissue slices.

## 3 Results

**FIGURE 3:**
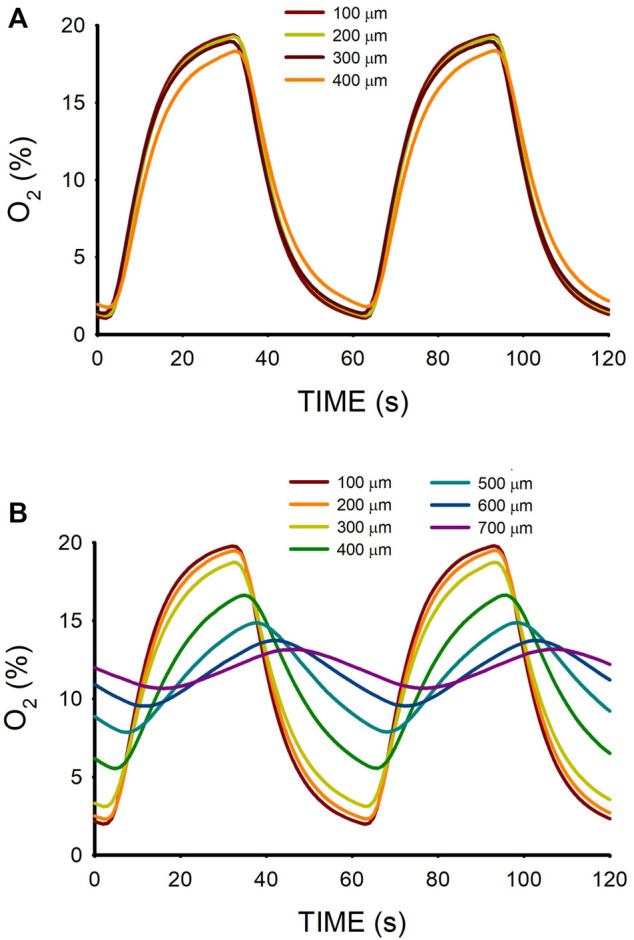
**(A)** Oxygen concentration measured at different positions (*z*; Figure 2A) within a lung extracellular matrix hydrogel of thickness W = 500 μm when the intermittent air circulating beneath the membrane was 20% O_2_ for 30 s 0% O_2_ for 30 s. **(B)** Same for hydrogel thickness W = 800 μm.

The time course of O_2_ concentration measured at different distances from the membrane (Figure 2A) in a W = 500-μm lung ECM hydrogel when subjected to intermittent hypoxia (20% O_2_-0% O_2_) at a rate of 60 events/h is shown in Figure 3A. For *z* = 100 μm, the maximum O_2_ concentration was virtually 20%, but the minimum was slightly greater than 0% O_2_ because of the slight drop in O_2_ concentration caused by diffusion across the PDMS membrane. As expected, increases in *z* decreased the amplitude of O_2_ concentration swings. However, the amplitude of O_2_ cycles was still considerable, even close to the top of the hydrogel (*z* = 400 μm). In contrast, when the thickness of the hydrogel was 800 μm, the amplitudes of O_2_ oscillations were considerably reduced as *z* increased (Figure 3B), with a maximum of 13.1% O_2_ and a minimum of 10.7% O_2_ for z = 700 μm.

**FIGURE 4:**
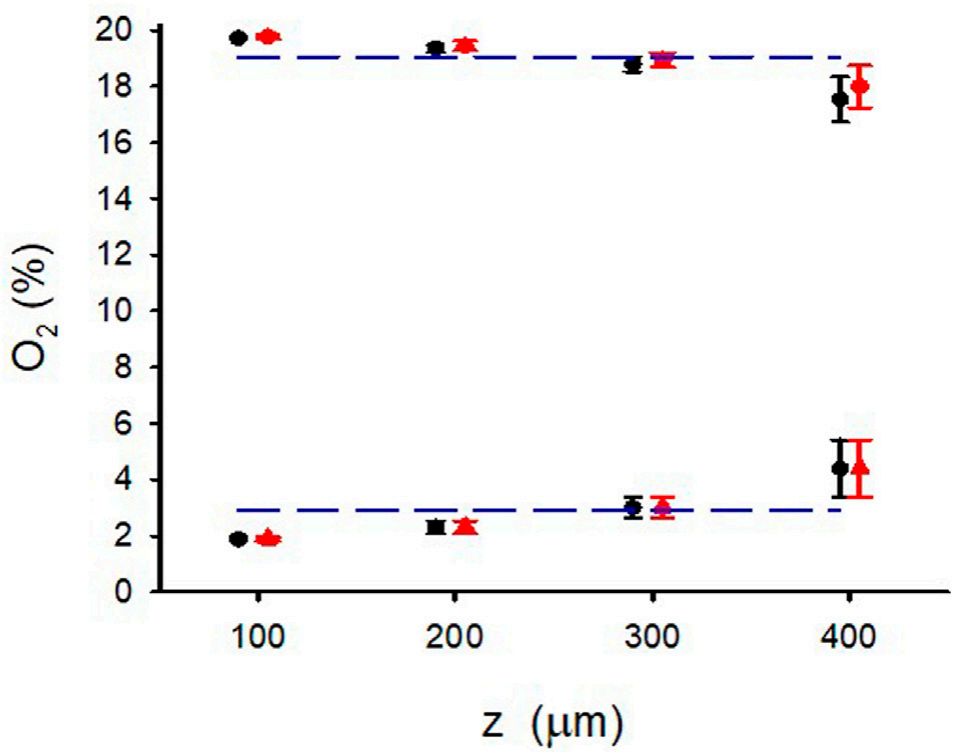
Maximum and minimum of O_2_ for different positions (*z*) in the W = 500 μm lung extracellular matrix hydrogel when the intermittent air circulating beneath the membrane was 20% O_2_ for 30 s 0% O_2_ for 30 s. O_2_ concentration was measured in 4 different random areas (data show the mean ± SD in each area) of two different hydrogel samples (red and blue symbols). Lines correspond to the mean corresponding to all values of *z.*

Figure 4 shows the excellent reproducibility observed (mean ± SD) of the maximum and minimum data shown in Figure 3A when the O_2_ measurements were carried out in 4 different random areas of two different W = 500-μm hydrogel samples. This figure also reflects that, despite the slight decrease in maximum and increase in minimum observed when *z* increased, the variance of minimum and maximum O_2_ concentrations across the hydrogel section was lower than 2% O_2_ around the corresponding mean value.

**FIGURE 5:**
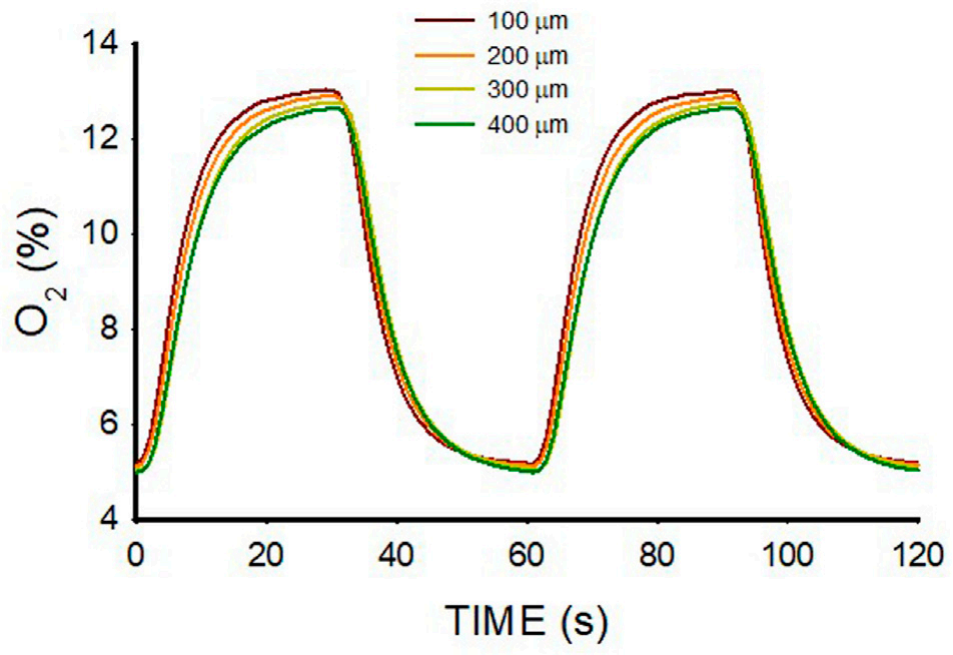
Oxygen concentration measured at different positions (*z*; Figure 2A) within a lung extracellular matrix hydrogel of thickness W = 500 μm when the intermittent air circulating beneath the membrane was 15% O_2_ for 30 s and 5% O_2_ for 30 s.

The range of O_2_ oscillations within the sample was readily modulated by modifying the O_2_ concentration of the gas circulating beneath the membrane. For instance, as shown in Figure 5, lower amplitude of intermittent hypoxic gas (15% O_2_–5% O_2_ in the gas beneath the membrane) subjected the hydrogel to oxygenation values close to the ones in the arterial blood perfusing tissues in patients with severe OSA (Figure 1A). The maximum and minimum values were similar over the hydrogel section (*z* from 100 to 400 μm), with maxima and minima ranging within .5% O_2_ and .2% O_2_, respectively.

**FIGURE 6:**
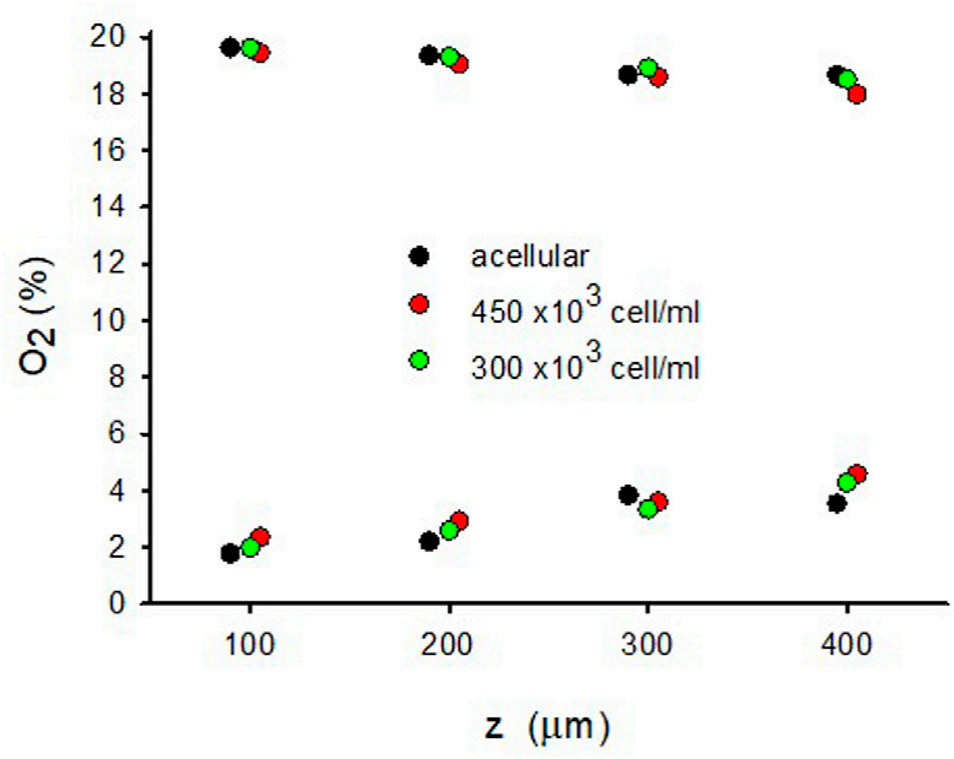
Maximum and minimum of O_2_ for different positions (*z*) in the W = 500 μm lung extracellular matrix hydrogel when the intermittent air circulating beneath the membrane was 20% O_2_ for 30 s 0% O_2_ for 30 s in an acellular hydrogel and when the hydrogel was seeded with two different concentrations of MSC.

3D culturing MSCs in the hydrogel did not significantly change the O_2_ diffusion across the sample as compared with the acellular hydrogel (Figure 3). Figure 6 shows the small differences in maxima and minima of O_2_ concentration observed in W = 500-μm lung ECM hydrogels in three cases: acellular and seeded with MSCs at two concentrations (300 and 450 × 10^3^ cells/mL).

**FIGURE 7:**
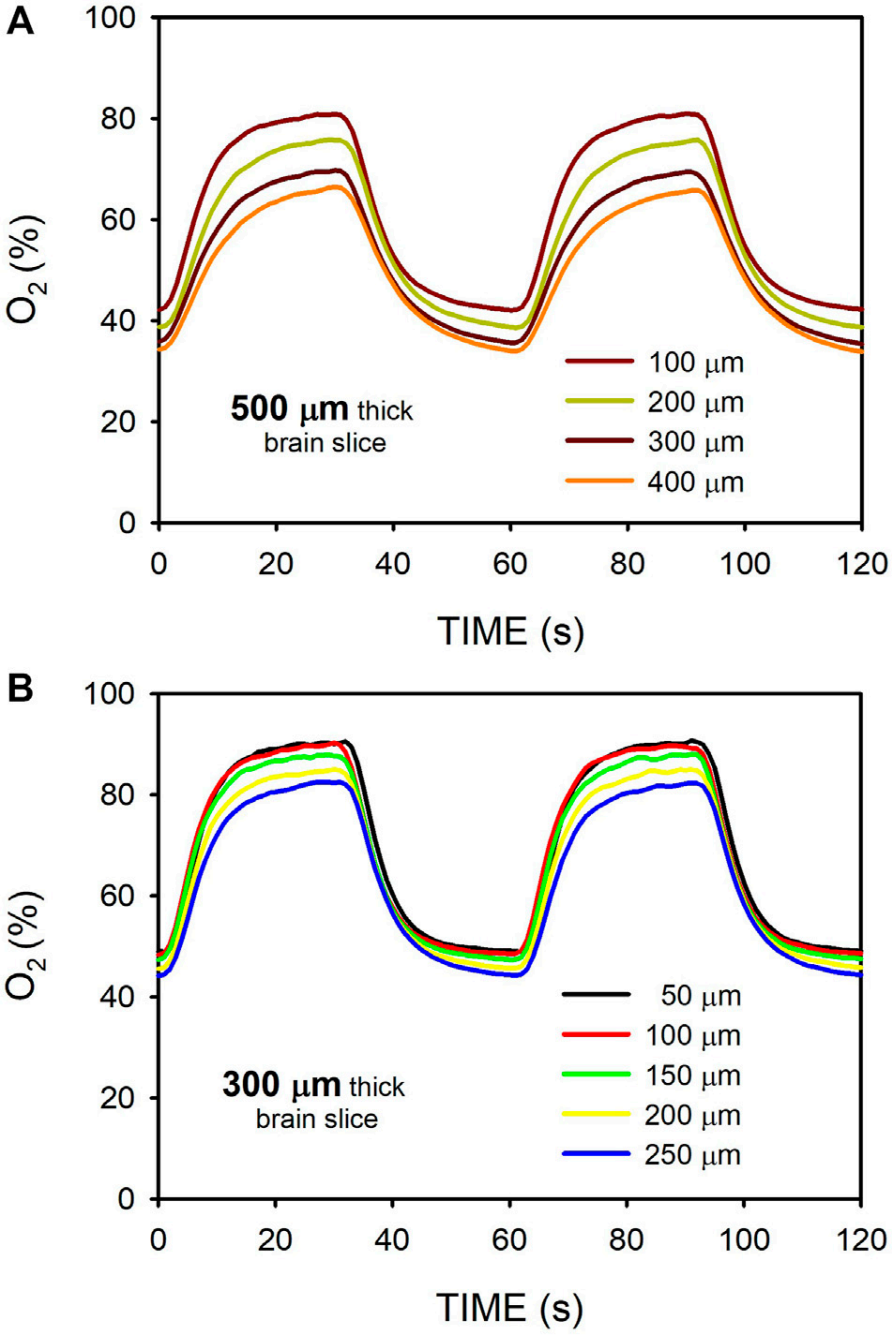
Oxygen concentration measured at different positions (Figure 2A) within mouse brain slices when the intermittent air circulating beneath the membrane was 95% O_2_ for 30 s and 50% O_2_ for 30 s. **(A)** O_2_ measured at the hippocampus of a 500-μm thick slice. **(B)** Same as in **(A)** for a slice thickness of 300-μm.

Figure 7 shows the O_2_ concentration recordings in mouse brain slices for 500-μm and 300-μm thicknesses. This figure further illustrates that changing the gas concentration flowing beneath the membrane (in this case 95%/50% O_2_) allowed modulation of the maximum and minimum of oxygenation in the sample. For W = 500-μm, O_2_ profiles were less homogeneous across the sample as compared with acellular (Figure 3) or MSC-cultured hydrogels (Figure 6). Indeed, the maximum - minimum of O_2_ were 80.8%–42.0% for z = 100 μm and 66.3%–34.0% for z = 400 μm (Figure 7A). In contrast, the intermittent hypoxia experienced across 300-μm mouse brain slices was nearly homogeneous, with maximum - minimum of O_2_ of 90.5%–49.0% for *z* = 50 μm and 82.3%—44.3% for *z* = 250 μm (Figure 7B).

## 4 Discussion

This study provides initial and compelling experimental evidence that it is possible to apply well-controlled fast intermittent hypoxia cycling to cultured cells in 3D environments mimicking severe sleep apnea, either when using an ECM hydrogel or when cells are maintained *ex vivo* in tissue slices.

In addition to its robust and reliable performance, one advantage of the experimental setting employed and tested in this study is its simplicity. Indeed, it is based on the fast diffusion of O_2_ through a thin membrane of PDMS, a material with a coefficient of diffusion for O_2_ (D ≈ 3.5 × 10^−5^ cm^2^/s) slightly higher than that of water (≈2.5 × 10^−5^ cm^2^/s) (Farré et al., 2018). Thus, the 3D diffusion time (∆t = L^2^/6·D) for O_2_ corresponding to a membrane with a thickness L = 100–200 μm is .5–2 s, a time much shorter than the periods of intermittent hypoxia in severe OSA (60 apneas/h). In this study, we used a commercially available PDMS membrane that was not particularly thin (165 μm). Therefore, employing thinner membranes (≤100 μm) as the ones also used in the bottom of cell culture wells (Campillo et al., 2016) could further fasten the transmission of O_2_ concentration changes to the 3D sample. Interestingly, as it does not require the use of complex microfluidic tools, this experimental setting can be easily employed by any cell biology laboratory: PDMS bottom wells are already commercially available or can be easily homemade (Campillo et al., 2016; Campillo et al., 2019). The other component required is a flow of intermittent hypoxic-normoxic gas beneath the membrane. It is of note that using a relatively expensive blender like the one employed in this work is not necessary. Indeed, the required square wave of O_2_ concentration can be achieved in a much easier and cheaper way by simply connecting the inlet of the well to two conventional sources of gas. For instance, compressed gas bottles (with a low-pressure regulator) can be alternatively connected to the well by a simple on-off timer (either by two low-cost perfusion pumps or a 3-way controllable valve).

The O_2_ microsensor was employed to characterize the specific levels and dynamics of hypoxia applied to the different 3D materials (hydrogels or tissue slices) and thicknesses. Therefore, once a given setting is defined, carrying out repeated and systematic experiments to study cell behavior should not require continued O_2_ measurements across all experiments. However, using the sensor is advisable when designing a specific experiment given that different hydrogels/tissues, and mainly their thickness, modify O_2_ transmission across the sample. It should be mentioned that an O_2_ microsensor device similar to the one employed in this study (or one based on Clark micro-electrode (Almendros et al., 2011) is not expensive (Otero et al., 2021). It is also worth noting that although we focused only on the application of intermittent hypoxia, the setting could easily be used for also applying intermittent hypercapnia which parallels intermittent hypoxia in OSA and is another challenge to normal cell response. Alternatively, both intermittent hypoxia and hypercapnia could be also implemented easily by a pre-mixed gas source, thereby reproducibly simulating naturally occurring events in patients with OSA (Imamura et al., 2019; Tripathi et al., 2019). However, we did not test intermittent hypercapnia because there is no available CO_2_ sensor with the size and time resolutions required for this application. Nevertheless, it can be anticipated that the setting would also work for intermittent hypercapnia, taking into account that the coefficients of diffusion for CO_2_ in PDMS (2.2 × 10^−5^ cm^2^/s) and water (2.1 × 10^−5^ cm^2^/s) are only slightly lower than for O_2_ (Hou et al., 2010; Kanehashi et al., 2012).

The results obtained in W = 500-μm hydrogels (Figure 3; Figure 5) show that O_2_ diffusion through the 3D scaffold was sufficiently fast to allow high-frequency intermittent hypoxia requiring 30-s for raising and decreasing times in O_2_ concentration across the 3D sample. Interestingly, ≈500 μm is a commonly used thickness for the research of 3D cell-seeded samples (De Hilster et al., 2020; Falcones et al., 2021; Marhuenda et al., 2022). The suitability of this thickness for optimal application of 60 hypoxic events/h was expected taking into account that the O_2_ coefficient of diffusion D in this type of hydrogel is slightly lower than that of water. Indeed, data from O_2_ diffusion studies in similar hydrogels (alginate, agarose, collagen, dense fibrin, reconstituted basement membrane (rBM/Matrigel)) indicate that D ranges around 75% of D in water (Ehsan and George, 2013; Colom et al., 2014). Also, it has been shown that in alginate and agarose hydrogels, the diffusion of small molecular weight species such as O_2_ is not significantly affected by the characteristics of the matrix (Li et al., 1996). Hence, assuming D = 1.87 × 10^−5^ cm^2^/s (75% of D in water), the diffusion time would be ∆t = 22 s which fits with the apparent time-constant of the quasi-exponential variation pattern of O_2_ concentration changes (Figure 3). However, it is remarkable that the diffusion rate and, thus, the amplitude of the high-frequency intermittent hypoxia cycles achieved largely depends on W, as illustrated by comparing the results of O_2_ cycling across two hydrogels that only differed in thickness. Whereas for W = 500 μm the transmission of O_2_ cycling was very satisfactory (Figure 3A), in the case of W = 800 μm the attenuation of O_2_ cycles amplitude was so apparent (Figure 3B) that no application of uniform intermittent hypoxia could be ensured for cells residing across the hydrogel. This considerable difference in results when comparing 500 μm and 800 μm thicknesses is not surprising given the quadratic dependence of the diffusion time ∆t on L (∆t = L^2^/6·D). Indeed, ∆t would be 57 s for L = 800 μm (as compared with 22 s for L = 500 μm), a time similar to the period of oscillation for 60 events/h.

Theoretically, the coefficient of diffusion D is not the only factor affecting the rate of O_2_ concentration change in cell-containing 3D scaffolds. Indeed, the rate of decreasing concentration *C*(*z,t*) depends on the coefficient of diffusion D according to the second Fick law and also on the cellular O_2_ consumption rate per unit volume of the material. Assuming that cellular O_2_ consumption follows the Michaelis-Menten kinetics:

$$\frac{\partial C\left(z,\;t\right)}{\partial t}=D\frac{\partial^{2}C\left(z,t\right)}{\partial z^{2}}-\frac{\;\rho_{cell}\cdot \;sOCR\;\cdot \;C\left(z,\;t\right)}{K_{m}+C\left(z,\;t\right)}$$ (1)

where *ρ*_cell_ is cell density, sOCR is the maximum rate of O_2_ consumption per single cell, and K_m_ is the half-maximum rate of O_2_ concentration (Magliaro et al., 2019). When considering Eq. 1 in the case of O_2_ unidirectionally flowing through a membrane-like hydrogel with thickness W, the rate of O_2_ consumption (V’_con_) per unit area within the volume can be estimated as

$${V’}_{con}=\frac{\rho_{cell}\;\cdot \;sOCR\;\cdot \;C_{mean}\;\cdot \;W\;}{\left(K_{m}+C_{mean}\right)}$$ (2)

where C_mean_ is the average O_2_ concentration across the construct. The diffusion flow (V’_dif_) across the membrane-like hydrogel is

$${V’}_{dif}=\frac{D\cdot ∆C}{W}=\frac{D\;\cdot \;\alpha \;\cdot ∆P\;}{W}$$ (3)

where ∆C is the difference in O_2_ concentration across the construct, *α* is the solubility of O_2_ in the material (assuming that of water *α* = 1.1 × 10^−6^ mol/(cm^3^ · atm)) and ∆*P* is the difference of O_2_ partial pressure across the membrane-like hydrogel. Therefore, the ratio between consumed and diffused O_2_ flows is

$$\frac{{V’}_{con}}{{V’}_{dif}}=\frac{\rho_{cell}\;\cdot \;sOCR\;\cdot \;C_{mean}\;\cdot \;W^{2}\;}{D\;\cdot \;\alpha \;\cdot ∆P\;\cdot \;\left(K_{m}+C_{mean}\right)}$$ (4)

Given the quadratic dependence of this ratio on W, the role played by O_2_ consumption is decreased as the hydrogel thickness is reduced. Indeed, assuming W = 500 μm, ∆*P* = 152 mmHg (corresponding to a difference from 20% O_2_ to 0% O_2_), and taking the parameters (D = 1.2 × 10^−5^ cm^2^/s, sOCR = 1.22 × 10^−16^ mol/(cell·s), and K_m_ = 4.1 × 10^−6^ mol/cm^3^) reported (Magliaro et al., 2019) for a collagen hydrogel seeded with hepatocytes with a cell concentration *ρ*_cell_ = 500 × 10^3^ cell/cm^3^, Eq. 4 results in a ratio V’_con_/V’_dif_ = .015, indicating that, in this case, the consumption of O_2_ can be neglected when compared with O_2_ diffusion. This conclusion remains valid when using the parameters measured for a cell concentration one order of magnitude higher (5 × 10^6^ cell/cm^3^) (Magliaro et al., 2019) or when considering cell types other than hepatocytes since, together with neurons, hepatocytes are the highest O_2_-consuming cells (McMurtrey, 2016). The fact that oxygen consumption is not relevant for applying O_2_ cycling in thin hydrogel samples is reflected by the results in Figure 6 showing virtually no differences between acellular and cell-seeded hydrogels.

The systematic O_2_ concentration measurements carried out in hydrogels have been complemented with preliminary data obtained when applying intermittent hypoxia to tissue slices. Figure 7 provides a proof-of-concept measurement in brain mouse slices having a thickness (300 and 500 μm) typical in studies using precision-cut tissue slices (Majorova et al., 2021). These results clearly suggest that, as in hydrogels, fast intermittent hypoxia can be applied to *ex vivo* tissue samples. We observed that for the same sample thickness of W = 500 μm O_2_ diffusion across the brain slices was slightly slower than across the hydrogels (Figures 3, 7), resulting in a less homogeneous distribution of intermittent hypoxia as *z* varied. Based on the two components in Eq. 1, two potential reasons may account for this observed difference. On the one hand, the diffusion coefficient for O_2_ in body tissues could be lower than in most usual hydrogels, as suggested by the few data available providing measured values of D for O_2_ in native tissues (MacDougall and McCabe, 1967). On the other hand, owing to higher cell density (*ρ*_cell_), the Eq. 1 component corresponding to the rate of O_2_ consumption by cells could be greater in native tissues than in artificial hydrogels. Regarding this particular point, it is interesting to note that when considering specific O_2_ consumption per cell (sOCR), brain tissue is most challenging for O_2_ transfer given the very high metabolic rate of its cellular components (McMurtrey, 2016). Although requiring further systematic analysis to characterize reproducibility in different types of *ex vivo* slices, the results obtained in brain samples suggest that similar or even better results could be obtained in 300–500 μm tissue slices from different organs (e.g., lung, liver, kidney, heart, pancreas, spinal cord or tumors (Wang et al., 2015; Stribos et al., 2016; Wu et al., 2019; Dewyse et al., 2021; Dimou et al., 2022)) particularly affected by end-organ OSA morbidity (Sánchez-de-la-Torre et al., 2013; Gileles-Hillel et al., 2016; Gozal et al., 2020; Brockmann and Gozal, 2022).

In conclusion, we have characterized an experimental approach, based on existing and easily available tools, that allows for controlled and precise application of high-frequency intermittent hypoxia both to 3D cell culture scaffolds such as ECM hydrogels as well as to precision-cut tissue slices. This approach, which is an advance on previous models investigating continuous hypoxia (Svanström et al., 2021), opens the door for the study of the specific noxious effects of intermittent hypoxia in the 3D-residing cells of the organs and tissues of patients with OSA under physiomimetic conditions.

## Data Availability

The raw data supporting the conclusion of this article will be made available by the author, without undue reservation.
